# Impact of Hyperhomocysteinemia and Different Dietary Interventions on Cognitive Performance in a Knock-in Mouse Model for Alzheimer’s Disease

**DOI:** 10.3390/nu12113248

**Published:** 2020-10-23

**Authors:** Hendrik Nieraad, Natasja de Bruin, Olga Arne, Martine C. J. Hofmann, Mike Schmidt, Takashi Saito, Takaomi C. Saido, Robert Gurke, Dominik Schmidt, Uwe Till, Michael J. Parnham, Gerd Geisslinger

**Affiliations:** 1 Fraunhofer Institute for Molecular Biology and Applied Ecology IME, Branch for Translational Medicine and Pharmacology TMP, Theodor-Stern-Kai 7, 60596 Frankfurt am Main, Germany; Natasja.Debruin@ime.fraunhofer.de (N.d.B.); Olga.Arne@ime.fraunhofer.de (O.A.); Martine.Hofmann@ime.fraunhofer.de (M.C.J.H.); mikeschmidt8@hotmail.com (M.S.); gurke@med.uni-frankfurt.de (R.G.); D.Schmidt@med.uni-frankfurt.de (D.S.); Michael.Parnham@ime.fraunhofer.de (M.J.P.); geisslinger@em.uni-frankfurt.de (G.G.); 2 Laboratory for Proteolytic Neuroscience, RIKEN Center for Brain Science, Wako, Saitama 351-0198, Japan; takashi.saito.aa@riken.jp (T.S.); saido@brain.riken.jp (T.C.S.); 3 Department of Neurocognitive Science, Institute of Brain Science, Nagoya City University Graduate School of Medical Sciences, Nagoya, Aichi 467-8601, Japan; 4 *pharmazentrum frankfurt*/ZAFES, Institute of Clinical Pharmacology, Goethe University, Theodor-Stern-Kai 7, 60590 Frankfurt am Main, Germany; 5 Former Institute of Pathobiochemistry, Friedrich-Schiller-University Jena, Nonnenplan 2, 07743 Jena, Germany; uwe.till.erfurt@web.de

**Keywords:** hyperhomocysteinemia, vitamin B deficiency, Alzheimer’s disease, amyloid beta-peptides, disease models, animal, memory and learning tests

## Abstract

Background: Hyperhomocysteinemia is considered a possible contributor to the complex pathology of Alzheimer’s disease (AD). For years, researchers in this field have discussed the apparent detrimental effects of the endogenous amino acid homocysteine in the brain. In this study, the roles of hyperhomocysteinemia driven by vitamin B deficiency, as well as potentially beneficial dietary interventions, were investigated in the novel *App^NL-G-F^* knock-in mouse model for AD, simulating an early stage of the disease. Methods: Urine and serum samples were analyzed using a validated LC-MS/MS method and the impact of different experimental diets on cognitive performance was studied in a comprehensive behavioral test battery. Finally, we analyzed brain samples immunohistochemically in order to assess amyloid-β (Aβ) plaque deposition. Results: Behavioral testing data indicated subtle cognitive deficits in *App^NL-G-F^* compared to C57BL/6J wild type mice. Elevation of homocysteine and homocysteic acid, as well as counteracting dietary interventions, mostly did not result in significant effects on learning and memory performance, nor in a modified Aβ plaque deposition in 35-week-old *App^NL-G-F^* mice. Conclusion: Despite prominent Aβ plaque deposition, the *App^NL-G-F^* model merely displays a very mild AD-like phenotype at the investigated age. Older *App^NL-G-F^* mice should be tested in order to further investigate potential effects of hyperhomocysteinemia and dietary interventions.

## 1. Introduction

After decades of research, there is still a huge unmet medical need for novel interventions to treat dementia-like disorders. In 2019 more than 50 million people were affected by dementia and the number could increase to about 152 million by 2050 [1]. Alzheimer’s disease (AD) is the most common type of dementia, accounting for 2/3 of all cases [2]. More than 400 failures in drug development during the last decades [3] have led to the consideration of alternative intervention options, e.g., repurposing, combinatory approaches and preventive treatments [4].

The complex pathology of the disease is characterized by several hallmarks, such as prominent extracellular amyloid plaques [5,6]. According to the amyloid cascade hypothesis, an alteration of amyloid-β (Aβ) metabolism is the central pillar of AD pathology and crucially influences and initiates other hallmarks [7]. In AD, initial pathologic processes progress decades before the first cognitive symptoms appear in patients, a stage entitled preclinical Alzheimer’s [8]. Disruptions in amyloid metabolism, as one of the first chronological hallmarks, potentially represent a relevant target for preventive interventions in AD.

In order to further elucidate disease mechanisms and identify novel treatment options, the group of Takaomi Saido at the RIKEN Center for Brain Science has developed a new generation of AD mouse models. These knock-in (KI) mice provide advantages compared to transgenic models, which are based on massive amyloid-β protein precursor (AβPP) overexpression with the result of artificial phenotypes due to overproduction of other AβPP fragments aside from Aβ. In the *App^NL-G-F^* model, the murine AβPP sequence is humanized and three mutations are introduced. Swedish (NL), Arctic (G) and Beyreuther/Iberian mutations (F) increase the total amount of Aβ and the Aβ42/Aβ40 ratio, show pro-inflammatory effects and finally result in a three times faster memory impairment [9].

Elevated levels of the endogenous amino acid homocysteine (HCys), called hyperhomocysteinemia, have been described as another hallmark of AD [10]. HCys is increased significantly in AD patients, whereas levels of different B-vitamins are reduced compared to controls [11,12]. A remaining question is whether hyperhomocysteinemia is merely a marker or whether it contributes causally to AD pathology, thereby providing options for therapeutic intervention. Some authors describe the role of plasma HCys as an independent risk factor for memory deficits and AD [13,14]. Consequently, B-vitamin supplementation as a HCys-modifying intervention was proposed previously [15]. According to Smith et al., B-vitamins lowered HCys levels and subsequently slowed the rate of brain atrophy and cognitive decline in patients [16,17]. However, a causal link between hyperhomocysteinemia and Alzheimer’s disease, called the “homocysteine hypothesis”, has been a source of controversy for years. Kennedy teased out the equivocal results of numerous studies in detail [18]. Several studies neither support an association of HCys with AD nor an improvement of cognitive performance by B-vitamin treatment [19,20]. Meta-analyses were conducted to assess this topic, challenging the homocysteine hypothesis and amelioration of cognitive functions by the use of folate and other B-vitamins [21,22]. In the context of an international consensus statement, researchers assessed the homocysteine hypothesis as being plausible and considered hyperhomocysteinemia a modifiable risk factor for dementia. Furthermore, they recommended considering polyunsaturated fatty acids (PUFAs) in addition to B-vitamins for future trials [23]. PUFAs such as docosahexaenoic acid (DHA) and eicosapentaenoic acid (EPA) are also suggested to be linked to AD pathology and HCys metabolism [24,25], i.e., elevated HCys impairs the formation of PUFAs and leads to a lower availability of PUFAs in the brain. B-vitamin treatment might only be successful when PUFA plasma concentrations are in the upper normal range [26]. A more recent systematic review points out that the evidence for nutrient supplementation remains limited and indicates that more research is needed to assess preventive measures in dementia [27].

**Figure 1:**
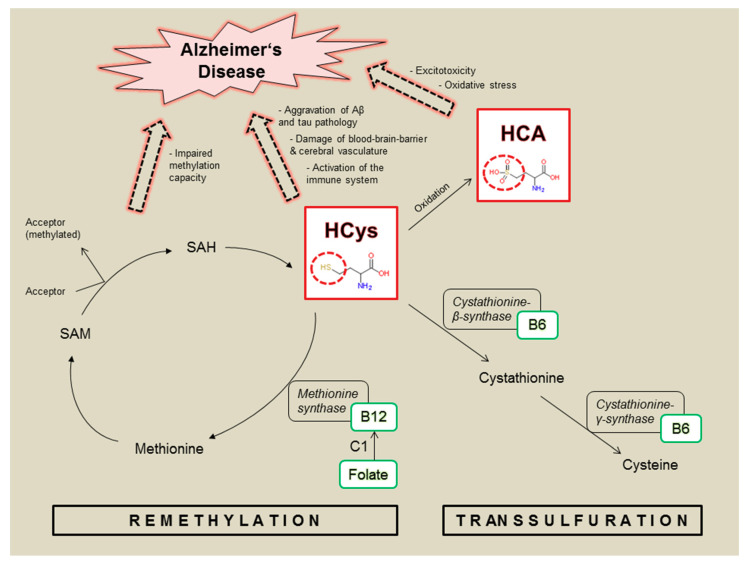
Homocysteine (HCys) and homocysteic acid (HCA): metabolic role and link to Alzheimer’s disease; involved enzymes (black boxes) linked to relevant B-vitamins (green boxes) functioning as coenzymes or methyl donor (C1); SAM = S-Adenosyl-L-methionine; SAH = S-Adenosyl-L-homocysteine.

Transsulfuration and re-methylation are major metabolic pathways for HCys (Figure 1), being dependent on an adequate supply of B-vitamins, particularly B6, B12 and folate [28]. As illustrated, the relevant B-vitamins play key roles in intrinsically decreasing HCys levels and therefore correlate negatively with HCys. Vitamin B12 and folate are crucial in providing methyl groups in the context of the re-methylation cycle, whereas the transsulfuration pathway depends on vitamin B6 as an essential enzymatic cofactor. Disturbed HCys metabolism (Figure 1) is likely to be linked to AD pathology by direct and indirect neurotoxic pathways [24]. Neurotoxicity is caused by excitotoxicity via N-methyl-D-aspartate receptor (NMDA) activation and by increased levels of reactive oxygen species promoting oxidative stress. Furthermore, excess HCys and subsequently a lack of methionine and S-Adenosyl-L-methionine (SAM), as well as elevated S-Adenosyl-L-homocysteine (SAH), are associated with a reduced methylation capacity and the inhibition of methylation reactions, which is suggested to exacerbate amyloid and tau pathologies in AD. Moreover, HCys results in an activated immune system, damages cerebral vessels and disrupts the blood-brain-barrier [24,29]. Both homocysteine and its oxidative metabolite homocysteic acid (HCA) are considered neurotoxic [30,31], but HCA is suggested to be the more potent species [32,33,34] and might contribute to dementia through oxidative stress and excitotoxicity by NMDA activation. Both mechanisms have been considered relevant for AD pathology [5,24].

The present exploratory animal study concentrates on the role of hyperhomocysteinemia, driven by vitamin B deficiency, in the context of AD. Therefore, we used the novel and not yet fully characterized *App^NL-G-F^* knock-in mouse as a model of the disease. The *App^NL-G-F^* mouse is expected to display a mildly impaired phenotype, simulating the very early preclinical period of AD pathology and thus should provide the possibility of assessing preventive interventions adequately. A versatile behavioral test battery should firstly assess potential deterioration of cognitive performance by hyperhomocysteinemia. Secondly, behavioral testing should clarify whether special diets enhance cognition and potentially could serve as preventive measures for AD. Here, we compared B-vitamins and PUFAs with a more complex micronutrient mixture similar to Fortasyn^®^ Connect [35]. HCys and HCA levels were measured in urine and serum using a validated LC-MS/MS method (liquid chromatography-tandem mass spectrometry) and the quantity of Aβ plaques in the brains was assessed.

## 2. Materials and Methods

A detailed description of all experimental procedures including the single behavioral testing systems, analytical methodologies and quality parameters of the current study can be found in Appendix A.

### 2.1. Animals and Experimental Diets

All experimental procedures were carried out in compliance with the ‘3R’ and in accordance with the Principles of Laboratory Animal Care (National Institutes of Health publication no. 86-23, revised 1985), the DIRECTIVE 2010/63/EU and the regulations of GV-SOLAS and were approved by the local Ethics Committee for Animal Research in Darmstadt, Germany (approval number: F152/1011; approval date: 31.07.2017). In the current study, 16 C57BL/6J wild type mice (WT) and 96 homozygous *App^NL-G-F^* knock-in (KI) mice, consisting equally of males and females, were included.

AIN93M chow served as a basis for the experimental diets and was modified, defining the different groups of *App^NL-G-F^* mice (Table 1). The exact composition of the diets is summarized in Table A1. Each mouse received four grammes of diet per day, except for the period of food restriction for males during the touchscreen PAL-task. Water was available ad libitum, except for the period of temporally conditioned water access for females during the IntelliCage experiment.

### 2.2. Behavioral Testing

**Figure 2:**
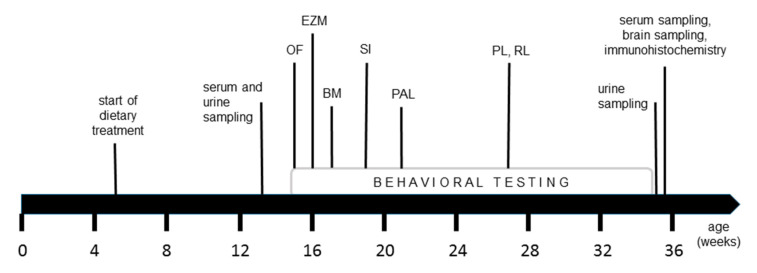
Time line of the study course; open field test (OF), elevated zero maze (EZM), Barnes maze (BM), social interaction test (SI), touchscreen paired associates learning (PAL) inclusive training phase, IntelliCage place learning task (PL) and reverse learning task (RL) inclusive habituation period; see Appendix A for detailed explanations of the single tests.

The testing battery we conducted consisted of diverse behavioral tests investigating different domains of cognition in the animals (Figure 2). At the age of 15 weeks, resp. 10 weeks on diet, the mice were first tested in the open field, followed by the elevated zero maze, Barnes maze and social interaction test. Finally, males were tested in a touchscreen task and females in the IntelliCage system.

Outcomes of every behavioral experiment were assessed automatically by camera or transponder detection. All experiments were performed between 8 a.m. and 3 p.m. during the light phase. After each trial, testing systems were cleaned with 70% ethanol to remove odors in the devices and to achieve comparable conditions for each animal.

### 2.3. Sample Collection

As illustrated in Figure 2, serum and 24-h urine of the mice were sampled after 8 and 30 weeks on experimental diets, resp. 13 and 35 weeks of age. The biological matrices were stored at −80 °C for subsequent analysis of HCys and HCA. At the end of the study, we euthanized all animals at the age of 35 weeks in order to harvest the brains. Brains were removed and post-fixed in 4% paraformaldehyde, followed by a stepwise dehydration, and embedding in paraffin. Ten µm thick sections were cut and mounted on glass slides for subsequent immunohistochemical analysis.

### 2.4. Biochemical and Immunohistochemical Analyses

The determination of HCA was performed as previously described in detail [36] using a combination of protein precipitation and solid phase extraction for sample preparation followed by an LC–MS/MS analysis applying a combination of a HILIC separation and tandem mass spectrometry. HCys was analyzed using protein precipitation in combination with reversed phase chromatography and tandem mass spectrometry.

Brain sections were immunohistochemically stained for amyloid-β peptides (Aβ) using an ABC/DAB protocol that is described in detail in Appendix A. After digitization of the sections, we analyzed the resulting images for the area of Aβ plaques in several regions of interest (ROI; Table A2), using ImageJ software.

### 2.5. Statistical Analyses

All experiments were statistically analyzed using IBM SPSS Statistics 25 (Ehningen, Germany). For each test, we conducted an outlier analysis in order to exclude extreme outliers (more than three times the interquartile range). Shapiro Wilk tests revealed whether Gaussian distribution could be assumed or not. Because of several data sets, which did not show a normal distribution, testing of statistically significant differences was computed by non-parametric Mann-Whitney-U-tests (comparison 1: C57BL/6J (group 1) versus *App^NL-G-F^* control (group 2); comparison 2: *App^NL-G-F^* control (group 2) versus *App^NL-G-F^* on special diets (groups 3–7)). A *p* value lower than 0.05 was considered statistically significant. Results were expressed as median ± interquartile range (IQR). Where applicable, medians were further compared to hypothetical medians using the non-parametric one-sample Wilcoxon signed rank test.

Graphical presentation was performed using GraphPad Prism 7 software (San Diego, CA, USA).

## 3. Results

### 3.1. Homocysteine and Homocysteic Acid

**Figure 3:**
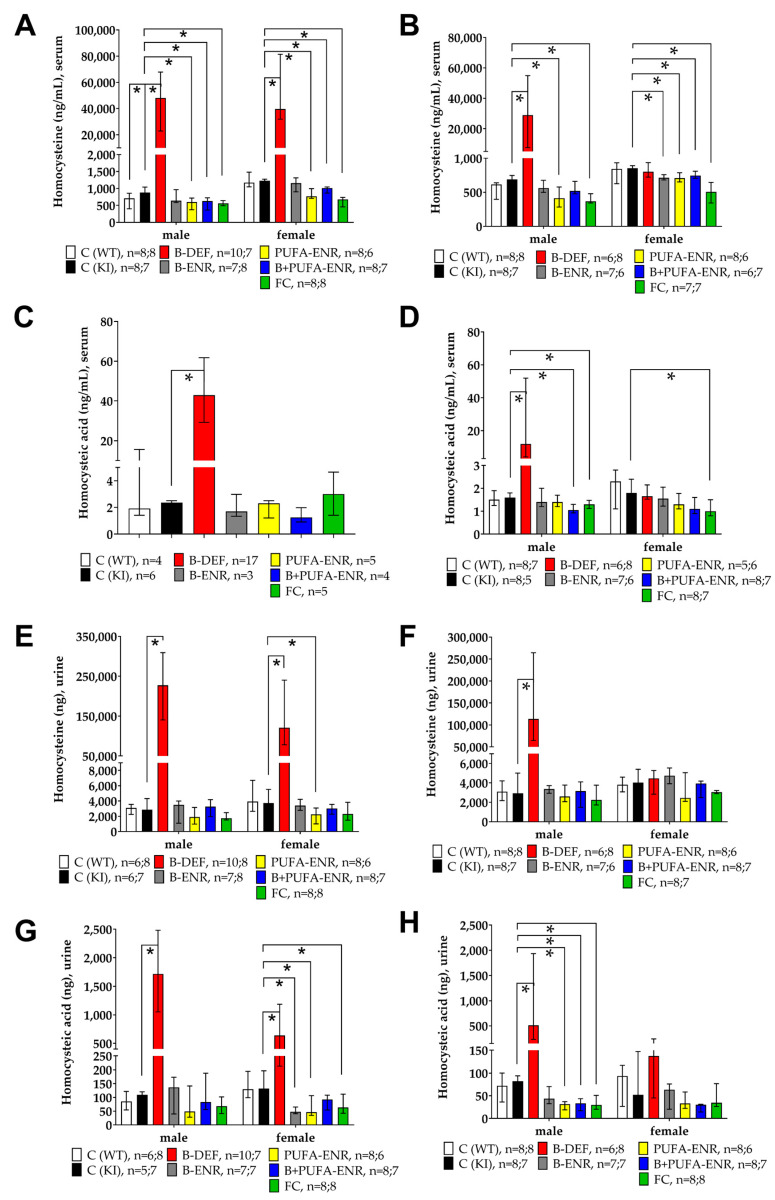
Homocysteine (HCys) and homocysteic acid (HCA) urine and serum levels in 13 and 35 weeks old C57BL/6J and *App^NL-G-F^* mice; 8 resp. 30 weeks on experimental diet; all samples analyzed by LC-MS/MS; data presented as median ± interquartile range (IQR); outliers beyond threefold IQR removed; *p* < 0.05 (Mann-Whitney-U-test) considered statistically significant (*). (**A**) HCys serum levels; 8 weeks on diet. (**B**) HCys serum levels; 30 weeks on diet. (**C**) HCA serum levels; 8 weeks on diet (males and females pooled); samples pooled for analytical method due to low volumes obtained by vena facialis puncture. (**D**) HCA serum levels; 30 weeks on diet. (**E**) HCys urine levels; 8 weeks on diet. (**F**) HCys urine levels; 30 weeks on diet. (**G**) HCA urine levels; 8 weeks on diet. (**H**) HCA urine levels; 30 weeks on diet.

LC-MS/MS analysis was performed in order to measure HCys and its oxidative metabolite HCA in serum and urine samples. Vitamin B deficiency resulted in an elevation of both HCys and HCA serum levels in males and females after 8 weeks on experimental diet (HCys male *p* < 0.001, female *p* = 0.001; HCA (pooled) *p* < 0.001) (Figure 3A,C). A consistent statistically significant difference between C57BL/6J wild type (WT) and *App^NL-G-F^* knock-in (KI) mice was not observed. Dietary interventions resulted in decreased serum levels of HCys (PUFA-ENR male *p* = 0.001, female *p* = 0.005; B+PUFA-ENR male *p* < 0.001, female 0.026; FC male & female *p* < 0.001). Serum samples had to be pooled for an adequate analysis of HCA because of low sample volumes obtained by vena facialis puncture (Figure 3C). Because of the resulting decreased number of observations, data are not depicted separately for males and females in this case. After 30 weeks on the diet, vitamin B deficient males remained significantly hyperhomocysteinemic (*HCys p* = 0.001; *HCA p* = 0.001), although to a lower extent, compared to 8 weeks on the diet, whereas females returned to baseline level due to the maintenance chow they received during the IntelliCage tasks. Analysis of 24-h urine samples delivered data that were largely comparable to the results from the serum samples. After 8 weeks on the diets (Figure 3E,G), both urinary HCys and HCA were significantly elevated because of the vitamin B deficient chow (HCys male & female *p* < 0.001; HCA male *p* = 0.001, female *p* = 0.035), whereas a genotype effect was not detectable. Experimental diets resulted in decreased amounts of HCys (PUFA-ENR female *p* = 0.014) and HCA (B-ENR female *p* = 0.001; PUFA-ENR female *p* = 0.022; FC female *p* = 0.040) in the urine compared to KI control mice. After 30 weeks on diets (Figure 3F,H), males deficient in vitamin B6, B12 and folate displayed elevated urinary amounts of HCys (*p* = 0.001) and HCA (*p* = 0.003), but to a lower extent compared to that after 8 weeks on the diets. Vitamin B deficient females showed equal quantities to the control groups due to the maintenance chow they had received during the IntelliCage tasks.

### 3.2. Open Field

**Figure 4:**
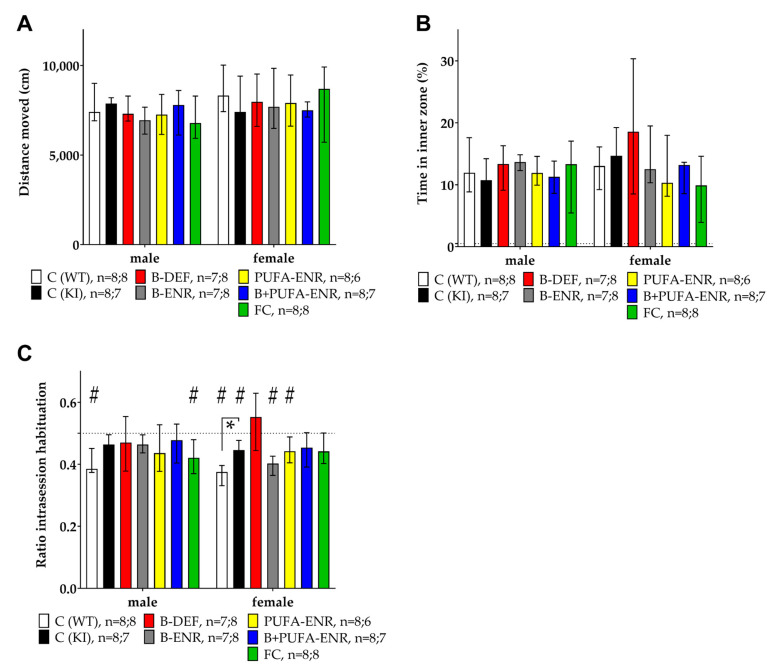
Open field test (30 min) in 15 weeks old C57BL/6J and *App^NL-G-F^* mice; 10 weeks on experimental diet; data presented as median ± IQR; outliers beyond threefold IQR removed; *p* < 0.05 (Mann-Whitney-U-test) considered statistically significant (*). (**A**) Total distance moved. (**B**) Percentage of time spent in inner zone. (**C**) Intrasession habituation expressed as ratio between the distance moved in the final time block divided by the sum of the final and the initial time block. (^#^) Ratio different from 0.5 (one-sample Wilcoxon signed rank test).

This behavioral test aimed to evaluate locomotion, anxiety, and habituation behavior of the mice during a 30-min session in the open field boxes. The total distance moved revealed no statistically significant differences (Figure 4A). Consequently, locomotion activity was not influenced by genotype or dietary intervention. The time the animals spent in the inner zone of the box, an indicator of anxiety, was not affected by genotype or diet (Figure 4B). As a third parameter, the amount of intrasession habituation was expressed by a habituation ratio (Equation (1)):

(1) ratio intrasession habituation = (5 min(final))/((5 min(final) + 5 min(initial)))

A ratio lower than 0.5 indicates habituation; a ratio of 0.5 means no change in activity, i.e., that no habituation occurred as in the case of groups 2–6 in males and groups 3 and 5–6 in females. Females fed with a vitamin B deficient chow displayed the least tendency to habituate; however, effects of experimental diets did not reach statistical significance in comparison to the KI control group. Female *App^NL-G-F^* control mice displayed a significantly lower level of habituation compared to the C57BL/6J WT control (*p* = 0.009), indicating an impact of the genotype (Figure 4C).

### 3.3. Elevated Zero Maze

**Figure 5:**
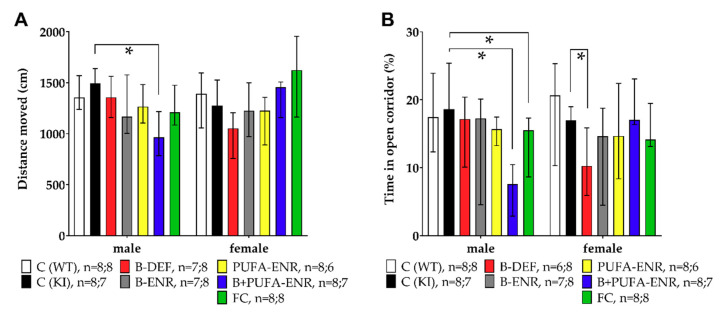
Elevated zero maze test (5 min) in 16 weeks old C57BL/6J and *App^NL-G-F^* mice; 11 weeks on experimental diet; data presented as median ± IQR; outliers beyond threefold IQR removed; *p* < 0.05 (Mann-Whitney-U-test) considered statistically significant (*). (**A**) Total distance moved. (**B**) Percentage of time spent in open corridors.

We tested anxiety behavior of each mouse for a session duration of 5 min. C57BL/6J WT and *App^NL-G-F^* KI control mice moved equal distances in the maze; only male *App^NL-G-F^* mice fed with a vitamin B and PUFA enriched diet moved less than *App^NL-G-F^* controls (*p* = 0.003) and thus displayed lower locomotion activity (Figure 5A). The time spent in the open corridors of the maze was an index for open space-induced anxiety in mice (Figure 5B). No genotype effect was observed between C57BL/6J and *App^NL-G-F^* mice, whereas different dietary interventions showed a reduction of cumulative time in open corridors. Particularly, male mice fed with the combination of PUFA and vitamin B enriched chow as well as with FC-like, spent significantly less time in the open corridors (*B+PUFA-ENR p* < 0.001; *FC p* = 0.021) and thus displayed increased anxiety. In females, a reduced time in the open corridors was observed in the vitamin B deficient group (*p* = 0.040) compared to KI control mice.

### 3.4. Barnes Maze

**Figure 6:**
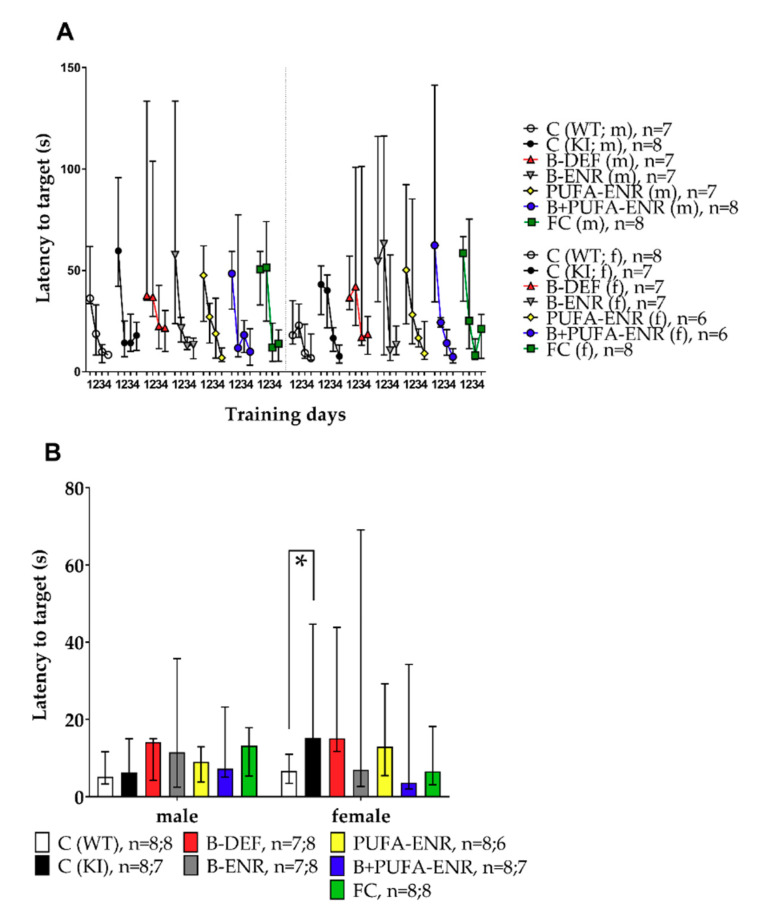
Barnes maze test in 17–18 weeks old C57BL/6J and *App^NL-G-F^* mice; 12–13 weeks on experimental diet; data presented as median ± IQR; outliers beyond threefold IQR removed; *p* < 0.05 (Mann-Whitney-U-test) considered statistically significant (*). (**A**) Latency to target hole; training days 1–4; 180 s per trial; acquisition phase; test on statistical significance carried out for day 4. (**B**) Latency to target hole; day 5; 90 s per trial; probe trial.

To investigate spatial memory and learning, the Barnes maze test was implemented in this study. In the first part of the test, the acquisition phase, the mice had to learn and remember the location of the escape box at the target hole. Figure 6A shows the latencies the mice needed to reach the target hole on subsequent days of training in the acquisition phase. The graph indicates a learning curve in every group. Tests on statistical significance were carried out for day 4 and revealed no differences at this stage of the test. In the probe trial on day 5 (Figure 6B), the reference memory of the previously learned target hole was tested. At this time, female *App^NL-G-F^* controls needed significantly longer to reach the target hole compared to the C57BL/6J WT control animals (*p* = 0.016). Vitamin B deficiency and corresponding hyperhomocysteinemia did not result in a worse performance at any stage of the Barnes maze test.

### 3.5. Social Interaction Test

**Figure 7:**
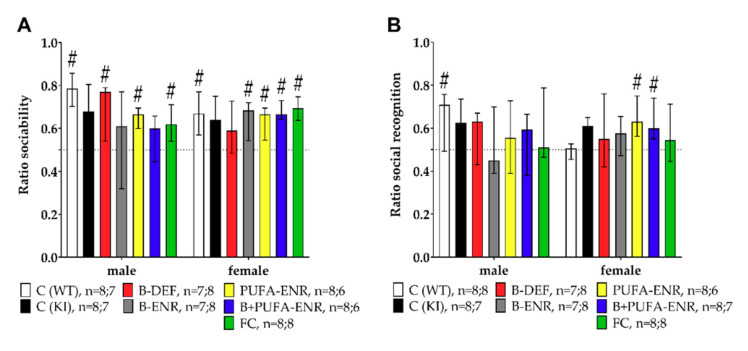
Social interaction test (25 min) in 19–20 weeks old C57BL/6J and *App^NL-G-F^* mice; 14–15 weeks on experimental diet; data presented as median ± IQR; outliers beyond threefold IQR removed; *p* < 0.05 (Mann-Whitney-U-test) considered statistically significant (*); (^#^) ratio different from 0.5 (one-sample Wilcoxon signed rank test). (**A**) Sociability expressed as the ratio between the contact time with the stimulus mice and the sum of the contact times with the stimulus mice and the empty cage. (**B**) Social recognition expressed as the ratio between the contact time with novel stimulus mice and the familiar ones.

Testing social behavior proceeded in two subsequent phases. At first, we assessed sociability, describing the curiosity of the animals towards the stimulus mouse in the testing system (Equation (2)) (Figure 7A).

(2) ratio sociability = (time social cage)/((time social cage + time empty cage))

No statistically significant difference was observed between C57BL/6J WT and *App^NL-G-F^* control animals. Experimental diets also had no impact on the social ability of the mice. Medians were statistically unequal to 0.5 except for group 2, 4 and 6 (males) and group 2 and 3 (females). A ratio of 0.5 means that contact times with the conspecific stimulus mouse and the empty cage were equal.

In the second phase of the test, we assessed the social recognition performance of the animals (Equation (3)) (Figure 7B).

(3) ratio social recognition = (time novel animal)/((time novel animal + time familiar animal))

As for sociability, neither genotype nor experimental diets had an influence on social recognition in the different experimental groups. In neither phase of the test did hyperhomocysteinemia aggravate the cognitive performance of the mice. Except for group 1 (males) and group 5 and 6 (females), medians of the other groups did not differ significantly from 0.5.

### 3.6. Paired Associates Learning (PAL) Task

**Figure 8:**
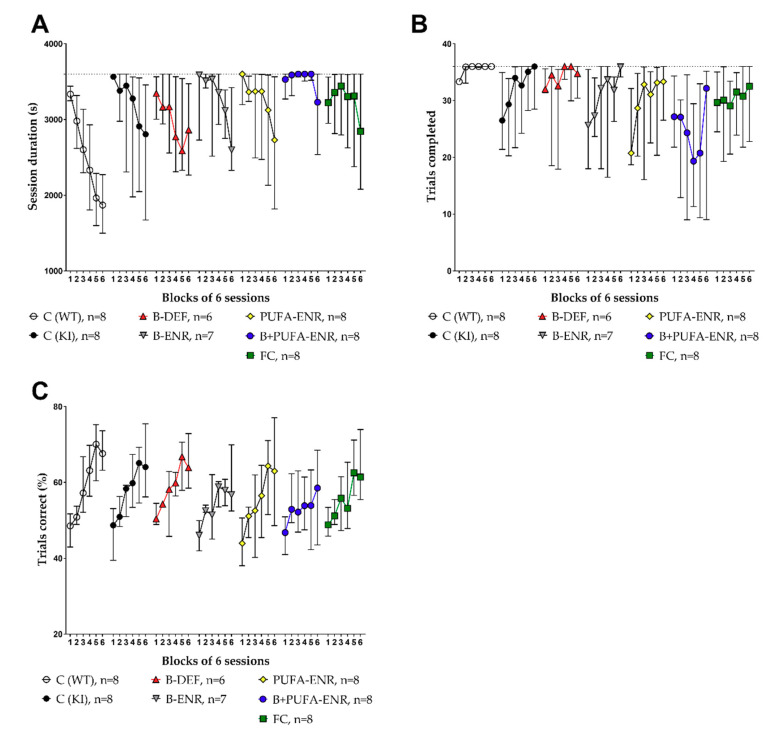
Touchscreen paired associates learning test (PAL) in 21–34 (incl. training phase) weeks old C57BL/6J and *App^NL-G-F^* mice; 16–29 weeks on experimental diet; only males; data summarized in blocks of 6 sessions and presented as median ± IQR; outliers beyond threefold IQR removed; test on statistical significance carried out for block 6. (**A**) Session duration: time (s) needed to complete 36 trials per session; maximum 3600 s. (**B**) Amount of trials completed per session; maximum 36 trials. (**C**) Percentage of correct trials per session.

The touchscreen PAL was used to assess potential cognitive impairment of the male mice (about five to eight months of age). Both the session duration and the number of trials completed per session, as well as the percentage of correct trials per session were analyzed (Figure 8). The resulting learning curves revealed no statistically significant difference in these parameters between C57BL/6J WT mice and *App^NL-G-F^* KI mice in the final phase of the test (block 6). Hyperhomocysteinemic *App^NL-G-F^* mice did not perform worse than *App^NL-G-F^* control mice. Other experimental diets also had no benefit on the cognitive abilities of *App^NL-G-F^* mice at this age. The C57BL/6J WT group showed a smaller variability in the touchscreen chambers in comparison to the *App^NL-G-F^* KI groups. This effect was particularly observed in the parameter trials completed (Figure 8B). Vitamin B deficient animals showed a tendency to perform better at the beginning of the test (trials completed, block 1) and thus did not display a learning curve like that of *App^NL-G-F^* control mice. However, no effects reached statistical significance in block 6. Animals fed with a vitamin B and PUFA combination diet did not reach the maximum number of trials per session. Therefore, the session duration scarcely also decreased over time in this group. The proportion of correct and incorrect trials was not affected.

### 3.7. Place Learning (PL) and Reversal Learning (RL) Task

**Figure 9:**
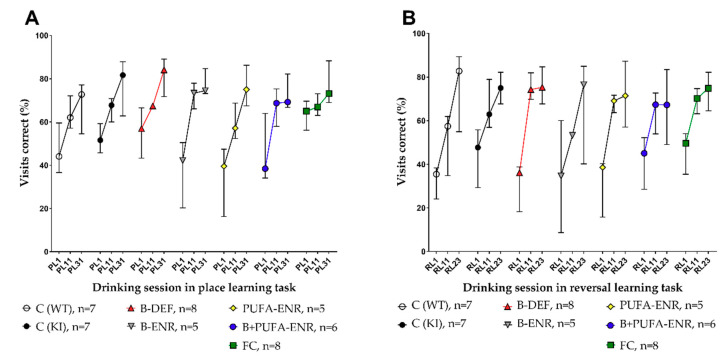
IntelliCage place learning (PL) and reversal learning (RL) task in 27–34 (incl. habituation period) weeks old C57BL/6J and *App^NL-G-F^* mice; 22–29 weeks on experimental diet; only females; data are shown for three points in time along the course of the task and presented as median ± IQR; outliers beyond threefold IQR removed; test on statistical significance carried out for the final point in time. (**A**) Percentage of correct visits during drinking sessions in the PL. (**B**) Percentage of correct visits during drinking sessions in the RL.

Learning and memory performance of the females at the age of about six to eight months was finally tested using two tasks in the IntelliCage system. We detected the visits of the mice to the drinking corners and analyzed the percentage of correct visits during the drinking sessions in the place learning (PL) and the reversal learning (RL) tasks. Three points in time along the course of the tasks are illustrated in Figure 9. Statistical analysis of the late phase of this course in both (Figure 9A) PL and (Figure 9B) RL (session 31; resp. 23) revealed no significant differences between *App^NL-G-F^* and age-matched C57BL/6J mice. In comparison to the *App^NL-G-F^* KI control group, none of the groups fed with experimental diets showed improved or impaired memory abilities.

### 3.8. Immunohistochemical Analysis

**Figure 10:**
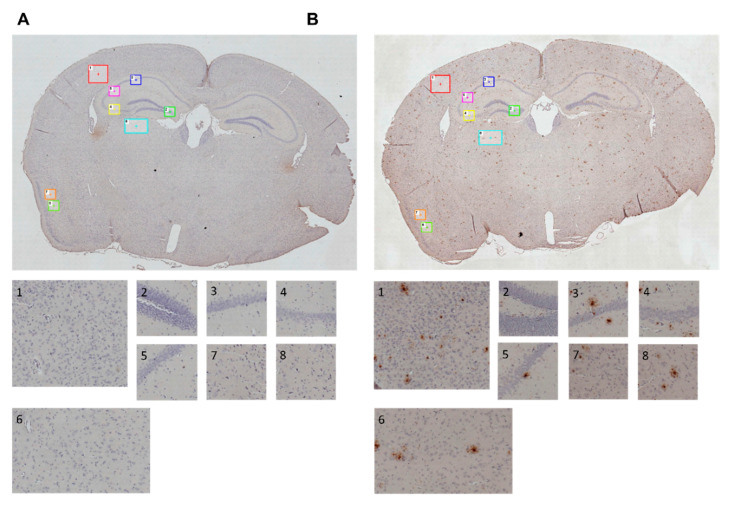
Immunohistochemically stained sections of mouse brains; regions of interest (ROI) in cortical and hippocampal areas are marked in whole brain images (100× magnification) and depicted separately for further semi-quantification of amyloid plaques; 35 weeks of age, 30 weeks on experimental diet. (**A**) Exemplary section of a C57BL/6J wild type animal. (**B**) Exemplary section of an *App^NL-G-F^* knock-in animal.

Brain sections of all animals were immunohistochemically stained and analyzed in order to semi-quantify the amount of amyloid plaques. For this purpose, we assessed the area (percentage) occupied by plaques in images of several regions of interest (ROI). The positions of the different cortical and hippocampal ROI (Table A2) are marked in Figure 10.

**Figure 11:**
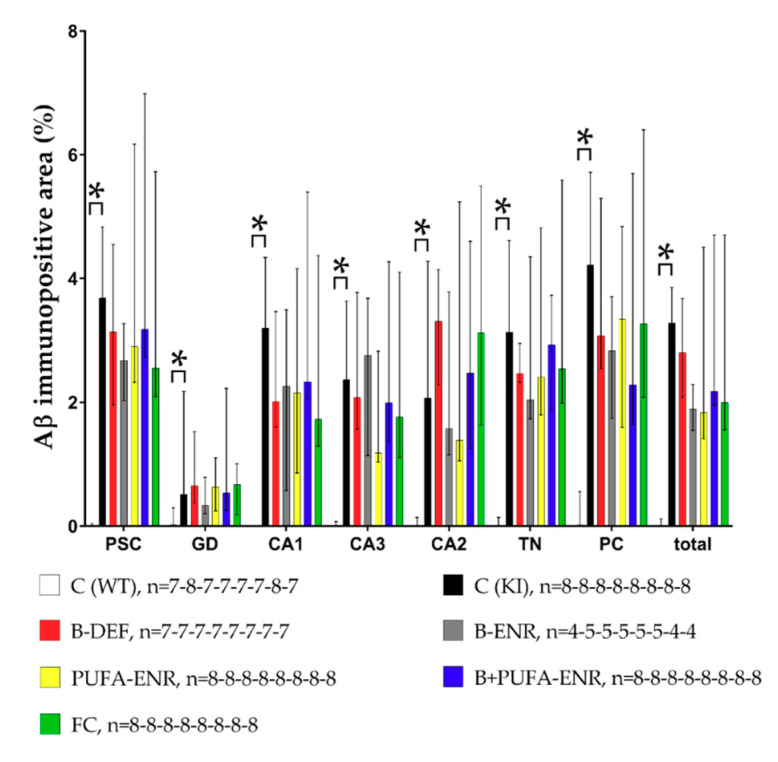
Semi-quantitative analysis of amyloid-β (Aβ) in immunohistochemically stained brain sections; results are shown for single regions of interest (ROI) and in total; 35 weeks of age, 30 weeks on experimental diet (males and females pooled); data presented as median ± IQR; outliers beyond threefold IQR removed; *p* < 0.05 (Mann-Whitney-U-test) considered statistically significant (*).

Figure 10 illustrates examples of brain sections of a C57BL/6J WT mouse and an *App^NL-G-F^* KI mouse. Aβ plaques, indicated by characteristic brown staining, occurred abundantly and diffusely in the brain sections of the KI animals (Figure 10B), whereas WT mice did not show any signs of Aβ deposition at all (Figure 10A). The differences in the Aβ burden between the C57BL/6J and *App^NL-G-F^* genotype, as well as a potential impact of the experimental diets, were further analyzed using ImageJ software. Semi-quantification of the Aβ burden confirmed a significant difference between WT and KI control groups (Figure 11) in all ROI (*p* < 0.001; *p* = 0.002; *p* < 0.001; *p* < 0.001; *p* < 0.001; *p* < 0.001; *p* < 0.001; *p* < 0.001).

There was no statistically significant difference in the plaque area between the diet groups and the *App^NL-G-F^* control group in the single ROI and in total. However, the immunohistochemical results indicate prominent plaque formation in all *App^NL-G-F^* groups at about 8 months of age.

## 4. Discussion

The current preclinical study investigated the impact of an induced hyperhomocysteinemia in the *App^NL-G-F^* knock-in mouse model for AD, as well as potentially preventive benefits of different micro-nutritional interventions. In order to characterize the phenotypes of the mice, we conducted a versatile behavioral test battery, accompanied by an analysis of HCys-/HCA levels and of the Aβ plaque burden. However, despite successful induction of prominent cerebral plaque deposition and hyperhomocysteinemia, merely subtle impairments were observed in the *App^NL-G-F^* mice.

C57BL/6J mice, a frequently studied mouse strain and background strain of the *App^NL-G-F^* knock-in (KI) model, served as an age-matched wild type (WT) control group in this study. Hence, results in these mice indicated a reference behavior and enabled subsequent assessment of the *App^NL-G-F^* genotype in the KI mice. In the open field, we focused on the intrasession habituation of the mice, which is one form of learning. Intrasession habituation describes a decreasing level of exploration of a new environment over time in a single session which can typically be detected in C57BL/6J mice [37]. This is in accordance with our finding that the habituation ratio in C57BL/6J was significantly lower than 0.5 and therefore indicated intrasession habituation. As expected, C57BL/6J mice demonstrated spatial learning and memory ability on consecutive days of training in the Barnes maze [38]. In a test for sociability and social recognition [39], C57BL/6J mice preferred to spent time with a conspecific (ratio sociability > 0.5) [40]. However, they did not prefer the novel conspecific in the second part of the test (ratio social recognition = 0.5). In the touchscreen PAL [41], male WT animals completed the maximum number of all 36 trials per session, accompanied by decreasing session duration. The increase in the percentage of correct trials is in accordance with observations in a similar study [42]. In the IntelliCage setup [43,44], learning curves indicated a constant learning effect in the female WT animals.

For several reasons, we decided to use an AβPP-based KI mouse model for AD in this study. Firstly, the novel KI models provide the advantage of not overexpressing AβPP in comparison to the more established transgenic models. Consequently, artificial phenotypes due to an overproduction of AβPP fragments besides the Aβ peptide should be avoided [9]. Secondly, an increased anabolism of Aβ levels is primarily a hallmark of hereditary- or early-onset AD [3]. Hyperhomocysteinemia, which is especially prominent in older people [45], is supposed to be a risk factor for AD [24]. Therefore, elevated HCys and HCA have been regarded as a hallmark of sporadic- or late-onset AD. The late-onset form affects the vast majority of AD patients [3]. By combining both the increased Aβ anabolism as a feature of hereditary AD and the detrimental effects of excess HCys as a feature of sporadic AD, we attempted to simulate cognitive decline more comprehensively. Thirdly, in order to investigate preventive treatments, it is mandatory to use a model displaying subtle phenotypes corresponding to a very early stage of the disease. According to a review by Zahs and Ashe, AβPP-based mouse models simulate the early phase of AD and thus are adequate for preventive interventions [46]. In the current study, a very subtle phenotype, i.e., very mild cognitive deficits, was observed. For each analysis, we compared C57BL/6J WT animals with *App^NL-G-F^* KI control animals. Both groups received the same control diet. KI mice displayed an impaired habituation behavior in the open field. Male mice of the two control groups habituated equally to the new environment, whereas females differed significantly. Data from the probe trial in the Barnes maze confirmed this finding: *App^NL-G-F^* KI mice needed longer to locate the former target hole than WT mice. As in the open field, this effect reached statistical significance only in females. Previous clinical studies suggest that a reduced cognitive reserve in women might explain the female vulnerability to develop a more severe phenotype of AD, a disorder affecting more women than men [47,48]. Other behavioral tests did not reveal differences caused by the KI genotype. However, data from the PAL test indicated an increased variance of results (higher IQR) in the *App^NL-G-F^* versus WT mice. WT animals showed a clearer performance curve with regard to the session duration and the number of trials completed along the course of the test, meaning that WT mice did not need as long as the KI mice to fulfil the 36 trials in a 1-h session. This enhanced efficiency might be the result of a higher motivation of the WT animals. Nevertheless, effects at the final stage of the test (block 6) did not indicate a significant impact of the genotype.

Other groups reported similar findings in *App^NL-G-F^* mice, indicating a very subtle phenotype. Two recent publications summarized these findings in tabular overviews, considering also sex and age of the mice of the included studies [49,50]. Latif-Hernandez and colleagues showed that the behavior of *App^NL-G-F^* mice was largely unaffected at the age of 3–10 months [51]. Similarities with our study can also be found in a publication by Whyte et al., who observed no differences between C57BL/6J and *App^NL-G-F^* mice in different cognitive tests at the age of 6 months [52]. Sakakibara and colleagues tested *App^NL-G-F^* mice at a higher age (15–18 months) and reported an intact learning ability but also recommended *App^NL-G-F^* as an AD model for preventive studies [53]. One year later, Jacob et al. observed neither consequences on cognitive performance in a touchscreen task nor age-dependent changes in a phase-amplitude coupling analysis, which was used as a measure of neurophysiological functioning, in 4.5 month old *App^NL-G-F^* mice. In accordance with our findings in the *App^NL-G-F^* model, these mice displayed a higher variability than WT control mice [42].

The question remains whether the KI mice were too young to display clear impairments. Further investigations are required to test the combination of the *App^NL-G-F^* genotype with our experimental diets in older mice. However, other groups detected significant cognitive deficits in the *App^NL-G-F^* model [9,49,50,54]. As summarized elsewhere [49], the majority of studies in the field investigated only male animals. Hence, a 1:1 comparison of these studies with our results comprising both sexes is difficult. Furthermore, a review of the topic described a relatively high level of variability in AβPP KI models between different laboratories [55]. Staining results of *App^NL-G-F^* brain sections showed prominent plaque deposition throughout the brain, as previously reported in similar studies [49,52], and thus indicate amyloid pathology as a central hallmark of early AD.

In order to investigate potentially detrimental effects of elevated HCys and HCA levels, one group of *App^NL-G-F^* mice received a special diet deficient in vitamin B6, B12 and folate. The resulting hyperhomocysteinemic state was confirmed in serum and urine prior to the start of behavioral tests. Our behavioral testing data obtained in the social interaction test, PAL and in the IntelliCages revealed no deficits in hyperhomocysteinemic mice and therefore do not support previous findings (e.g., [56]). The open field test and Barnes maze indicated subtle deficits in habituation behavior and spatial learning and memory, but these effects did not reach statistical significance. Only the elevated zero maze revealed an increased anxiety in hyperhomocysteinemic females. This observation might be of translational relevance, because anxious behavior is also one aspect of the AD phenotype [57]. Various preclinical studies in the field indicate a significant impact of hyperhomocysteinemia on plaque burden [58,59]. Other groups reported no such effects, which is in accordance with our immunohistochemical results in the *App^NL-G-F^* model [60,61]. In conclusion, despite severely elevated levels of HCys and HCA over a longer period of their life span, *App^NL-G-F^* mice showed neither a modified plaque burden nor significant cognitive deficits due to hyperhomocysteinemia. A majority of preclinical data published in the field indicate behavioral deficits in animal models caused by increased HCys (e.g., [56,59,62]). However, we assume that the evidence might be biased to some extent. On the one hand, behavioral data obtained in transgenic models based on massive AβPP overexpression might be somewhat artificial because of an overproduction of other AβPP fragments aside from Aβ [9]. It should also be considered that negative results are often not published, although equally important as positive results. The publication bias, meaning the reduced publishing of negative or null results, is not restricted to the field of AD research, but is rather a general problem [63].

Hyperhomocysteinemia is referred to as a hallmark of AD [10], but its impact on the disease is still under discussion. From a translational point of view, this experimental group simulates the portion of elderly people who are deficient in B-vitamins [64]. Preclinical evidence [65] and clinical evidence [45] confirm an age-related elevation of HCys levels. An impaired vitamin status is one reason amongst others for hyperhomocysteinemia in the elderly [66]. In the present study, the lack of vitamin B6, B12 and folate in combination with 1% sulfathiazole sodium to inhibit bacterial folate synthesis in the gut [58], led to a “severe” hyperhomocysteinemic state, according to a classification used in other publications [67]. Consequently, our vitamin B deficient mice displayed high HCys serum concentrations (45,760 ng/mL ≈ 339 µmol/L) in comparison to our *App^NL-G-F^* KI control (1054 ng/mL ≈ 8 µmol/L) and in comparison to elevated HCys levels in similar studies (e.g., [56,62,68]). Fuso and colleagues also reached high plasma total HCys (>400 µmol/L ≈ 54,000 ng/mL) in their study with TgCRND8 mice and explained the relatively high levels by not fasting the mice before sacrifice and by inhibiting both the re-methylation and the transsulfuration pathway [58].

Vitamin B deficient chow resulted in ~50 fold higher serum and urinary HCys and ~10–20 fold higher serum and urinary HCA compared to animals fed with control diet for 8 weeks. About 0.1% of HCys molecules were oxidized to HCA in serum (42.9 ng/mL ≈ 0.23 µmol/L) and excreted in urine (1184 ng) in 24 h. Only free HCys can be oxidized to HCA, which is suggested to be the main neurotoxic species [32,33,34]. In the current study, we did not measure the free form but the levels of total HCys by adding a reduction step (TCEP-solution) in the analytical method. In vivo, most HCys molecules are protein-bound or dimerized; only about 1% are available in the free thiol form [12]. Hasegawa et al. reported cognitive impairment in transgenic 3xTg-AD mice, triggered by elevated HCA in the brain [69].

We also investigated other experimental diets besides the vitamin B deficient chow discussed above. Group 4 received a vitamin B enriched diet containing a particular high content of folate, B6 and B12 compared to both the control diet and the FC-like diet. The goal of this diet was to investigate whether an additional increase, specifically of B-vitamins, in comparison to the FC-like diet could provide further benefits in the outcome of the study. Therefore, the difference in B-vitamin contents should simulate a potentially different effectiveness between FC (Souvenaid^®^) and existing higher dosed vitamin B preparations as human treatment options. In accordance with a recent international consensus statement [23], PUFAs (DHA + EPA) have been suggested to be beneficial for cognitive functioning in general and might be additionally linked to AD pathology [25,70]. Because single nutrient intervention studies often failed to show beneficial effects on cognitive function, it has been suggested that it might be important to investigate combinatory approaches [35]. For this purpose, we combined the high content vitamin B enrichment with the supplementation of PUFAs (group 6). Finally, group 7 received the FC-like diet, a complex mixture of ingredients (Table A1), which we implemented due to positive previous findings (e.g., [35,71]).

Supplementation of B-vitamins and PUFAs, as well as combinatory approaches and the FC-like mixture, were capable of lowering HCys and HCA below the levels of the *App^NL-G-F^* control mice fed with a standard rodent chow. However, by taking both sampling points (8 and 30 weeks on diet) as well as behavioral testing data into consideration, results appear inconsistent. In the open field, anxiety-related behavior did not differ between groups fed with B-vitamins, PUFAs or a mixture and *App^NL-G-F^* control animals. However, the elevated zero maze revealed increased anxiety in males fed with the combination diets. Especially the mice supplemented with both B-vitamins and PUFAs were more anxious and stayed in the closed corridors of the zero maze, but it has to be emphasized that these mice also displayed a reduced locomotion activity during the test. In the Barnes maze, experimental chow did not affect latencies to target at day 4 of training. Other researchers too did not observe benefits of PUFA-supplementation in cognitive tasks [72]. We confirmed the lack of dietary effects on cognitive performance in the social interaction test, the IntelliCage and PAL. Although not significant in the final block of 6 sessions, the session duration and trials completed indicate a worse learning curve for group 6 (B+PUFA-ENR) in the PAL test. This might be due to a lack of motivation in these mice receiving a high number of vitamins and PUFAs, which possibly lowered their affinity to the milk reward in the PAL task. One reason could be that the food restriction was not strict enough for this group. The FC-like diet did not prove beneficial in any test in comparison to the control chow. This is in accordance with some clinical studies, which do not support the benefit of the FC diet and thus indicate equivocal evidence [73,74]. In conclusion, the beneficial tendencies we observed did not mostly reach statistical significance in behavioral tests and biochemical-/immunohistochemical analyses and consequently do not suggest a clear beneficial effect of B-vitamins or PUFAs in this mouse model at the investigated age and diet duration. It is important to question here whether it is possible to observe amelioration through dietary intervention when merely a subtle behavioral deficit is induced in the KI mouse model.

Overall, this mouse model, simulating amyloid pathology without AβPP overexpression, merely displays a very mild phenotype despite massive cerebral Aβ deposition at the age of 35 weeks. The amyloid hypothesis has been questioned frequently because of the disappointing track record in clinical trials of drugs that target Aβ despite decades of extensive research in the field [7,75]. In addition, in some cases, substantial plaque deposition does not even cause dementia-like symptoms [76]. However, the window for potentially preventive measures is limited to an early stage of AD, where cerebral amyloidosis remains the central hallmark of the pathology [3]. Despite all criticism of the amyloid hypothesis, beneficial effects were recently observed using the human anti-Aβ monoclonal antibody aducanumab [77], confirming a causal role of Aβ in AD pathogenesis.

## 5. Conclusions

The current study only indicates a mild hyperhomocysteinemia-driven exacerbation of the AD-like phenotype, simulated in the *App^NL-G-F^* knock-in mouse model. Dietary interventions consisting of B-vitamins and/or PUFAs as well as the FC-like diet as a complex micronutrient mixture were unable to modify cognitive performance in this mouse model for AD. Neither the B-vitamin deficient diet, resulting in elevated HCys and HCA levels, nor the potentially beneficial diets affected the amount of plaque deposition in the brain. In comparison with the age-matched C57BL/6J wild type control group, *App^NL-G-F^* control mice displayed merely subtle behavioral deficits at the investigated age. Further investigations should clarify whether the *App^NL-G-F^* genotype and the experimental diets have an impact in older animals.

## Figures and Tables

**Table 1:** Details of the experimental groups.

Group Number	Genotype	Diet	Abbreviation
1	C57BL/6J wild type	Control	C (WT)
2	*App^NL-G-F^* knock-in	Control	C (KI)
3	*App^NL-G-F^* knock-in	Vitamin B deficient	B-DEF
4	*App^NL-G-F^* knock-in	Vitamin B enriched	B-ENR
5	*App^NL-G-F^* knock-in	PUFA supplemented	PUFA-ENR
6	*App^NL-G-F^* knock-in	Vitamin B enriched and PUFA supplemented	B+PUFA-ENR
7	*App^NL-G-F^* knock-in	Fortasyn^®^ Connect-like	FC

## Appendix A

### Animals

Wild type (WT) mice were purchased from Charles River Wiga GmbH (Sulzfeld, Germany), whereas the knock-in (KI) mice were kindly provided by the RIKEN Center for Brain Science (Saitama, Japan) on a C57BL/6J background and further bred at mfd Diagnostics GmbH (Wendelsheim, Germany). After their arrival at our facility at the age of four weeks, the animals were chipped with subcutaneous transponders to facilitate identification and to enable the IntelliCage task. Furthermore, additional genotyping via polymerase-chain-reaction analysis was carried out to ensure the adequate genetic background of each animal. Their allocation to the home cages was in a randomized order. All animals were housed in groups of two mice per cage (Green Line, Tecniplast, Hohenpeissenberg, Germany). In the maintenance room, constant temperature (mean: 22.7 °C) and humidity (mean: 48.6%) conditions as well as a 12/12 h dark/light cycle were provided. The pathogen-free status of the maintenance room was regularly monitored using sentinel mice. After an acclimatization phase of one week, the mice were allocated randomly to the experimental groups based on different diets. Body conditions scores were monitored, and the mice were weighed every week.

### Experimental Diets

The composition of the FC-like diet was oriented towards the work of Jansen et al. All diets containing PUFAs were stored at −20 °C to minimize oxidation [35]. Due to coprophagia (the ingestion of fecal matter) in mice, the vitamin B deficient diet additionally contained the antibiotic sulfathiazole sodium (Sigma-Aldrich, Taufkirchen, Germany) to prevent bacterial folate synthesis in the gut [58]. All experimental diets were purchased from Ssniff-Spezialdiäten GmbH (Soest, Germany).

**Table A1:** Composition of the experimental diets.

	Control	B-DEF	B-ENR	PUFA-ENR	B+PUFA-ENR	FC
Casein	140.0	140.0	140.0	140.0	140.0	140.0
Corn starch	355.6575	345.6920	355.4795	355.6575	355.4795	328.3386
Maltodextrin	155.0	155.0	155.0	155.0	155.0	155.0
Sucrose	100.0	100.0	100.0	100.0	100.0	100.0
Dextrose	100.0	100.0	100.0	100.0	100.0	100.0
Cellulose	50.0	50.0	50.0	50.0	50.0	50.0
Mineral premix	35.0	35.0	35.0	35.0	35.0	35.0
Vitamin pre-mix (w/o B-vitamins)	10.0	10.0	10.0	10.0	10.0	10.0
Soybean oil	19.0	19.0	19.0	——	——	——
Coconut oil	9.0	9.0	9.0	11.3	11.3	11.3
Corn oil	22.0	22.0	22.0	18.7	18.7	18.7
Fish oil (eicosapentaenoic acid/docosahexaenoic acid = 1:4)	——	——	——	20.0	20.0	20.0
L-Cystine	1.8000	1.8000	1.8000	1.8000	1.8000	1.8000
Tert-butylhydroquinone	0.0080	0.0080	0.0080	0.0080	0.0080	0.0080
Choline bitartrate, 41%	2.5000	2.5000	2.5000	2.5000	2.5000	2.5000
Pyridoxine-HCl (Vit. B6)	0.0070	——	0.1000	0.0070	0.1000	0.0398
Cyanocobalamin, 0.1% (Vit. B12)	0.0250	——	0.1000	0.0250	0.1000	0.0600
Folic acid, 80%	0.0025	——	0.0125	0.0025	0.0125	0.0100
Sodium selenite • 5 H_2_O, 30%	——	——	——	——	——	0.0036
Choline chloride, 43% Choline	——	——	——		——	6.9700
Ascorbic acid, 100% (Vit. C)	——	——	——	——	——	1.6000
DL-a-tocopheryl acetate, 50% (Vit. E)	——	——	——	——	——	4.6500
Uridine monophosphate disodium (24% H_2_O)	——	——	——	——	——	10.0000
Soy lecithin	——	——	——	——	——	4.0200
Sulfathiazole sodium	——	10.0000	——	——	——	——
Sum	1000	1000	1000	1000	1000	1000

### Open Field

Besides its value as a test for locomotor activity and anxiety, the open field task provides information on habituation as a form of learning [37]. For this purpose, each mouse was placed into the center of a 28.5 × 29.8 cm box (in-house manufactured, Fraunhofer IME, Schmallenberg, Germany). Animals were allowed to explore the new environment for 30 min. Total distance moved and percentage of time spent in the inner zone of the box were automatically detected by camera tracking and corresponding EthoVision XT 13 software (Noldus, Wageningen, The Netherlands). Data were analyzed additionally for time blocks of 5 min.

### Elevated Zero Maze

To investigate anxiety related behavior [78], we placed each animal into the open corridor of a 60 cm diametric elevated zero maze (Ugo Basile SRL, Gemonio, Italy) for a duration of 5 min. The maze consisted of two open and two closed 5 cm wide corridors. Besides the time spent in the open corridors, the total distance moved by the mice was automatically detected by camera tracking and corresponding EthoVision XT 13 software.

### Barnes Maze

The Barnes maze test is a common tool to measure spatial learning and memory [38] in AD mouse models, based on the aversion of mice to bright open spaces. We particularly preferred the Barnes maze over the Morris water maze, since it presents a less aversive alternative [79]. The apparatus (Ugo Basile SRL, Gemonio, Italy) consisted of a circular surface (diameter 100 cm) with 20 holes at the edge and an escape box positioned below one of the holes. There were four different visual cues positioned around the maze. The task required the mouse to localize the escape hole and enter the box. Initially, we transported each animal to the center of the maze in an opaque vessel to prevent an orientation before the start of the trial. The procedure was divided into two phases. First, in the acquisition phase, each mouse was subjected to two trials per day for four days (3-min limit per trial; inter-trial interval 15–30 min). The trials ended when either the mouse entered the escape box or when a duration of 180 s was over. On day 5, animals were subjected to a probe trial (90 s). During this phase, the escape box was not available anymore. Latencies to the target hole (acquisition & probe) were automatically detected by camera tracking and corresponding EthoVision XT 13 software.

### Social Interaction Test

This method enables the assessment of sociability and social recognition in mice [39]. For this purpose, a three-chamber cage consisting of a central chamber and two lateral compartments (Noldus, Wageningen, Netherlands) was used. The lateral compartments included sex-matched stimulus mice in separate acrylic rod cages, which allowed social interaction without direct contact. Test animals explored the setup during three consecutive phases. During the first time block of 5 min, the mice were allowed to explore only the middle chamber. As a next step, we opened the dividers to the lateral compartments and placed a stimulus mouse into one of the rod cages (social cage). The second rod cage remained empty. The experimental mouse had a period of 10 min to explore the whole three-chamber cage and to interact with the unknown stimulus mouse. For the next 10 min, we placed an additional unknown stimulus mouse into the second rod cage. The cumulative contact time with the familiar and non-familiar conspecific was automatically detected by camera tracking and corresponding EthoVision XT 13 software.

### Paired Associates Learning (PAL) Task

The ability of visuospatial associative learning was tested in males in the touchscreen PAL (touchscreen and corresponding Abet II Touch 18.7.6 software: Campden Instruments, Loughborough, UK and Lafayette Instrument Company, Lafayette, IN, USA). The task requires a lot of training, but is also a valuable tool in terms of translational cognitive research due to its similarities with the human CANTAB [41,80]. Based on the Bussey-Saksida method, animals initially were habituated to the touchscreen chambers during different pre-training phases. After completion, mice were introduced to the proper PAL task. Here, two objects were shown in two spatial locations on the screen. In each trial, only one correct association of object and location was presented, and the animal had to detect it via nose poke. As a result, a reward was delivered automatically (sugared condensed milk, 7 µL, Hochwald Foods GmbH, Thalfang, Germany). Incorrect responses were followed by an aversive light stimulus (5 s time-out period). After an inter-trial interval (20 s), the next trial was initiated by the mouse. A session ended when either 36 trials were completed, or 60 min ran out. The animals were food restricted through the whole experiment with the aim of reducing body weights to about 90% of the baseline weight before the test. This should enhance the motivation of the mice to collect the reward after each correct trial. Animal weights were monitored three times a week. For the assessment of the 36 sessions of the PAL task, the parameters’ session duration, trials completed, and percentage of correct trials were analyzed. The procedure was highly standardized and the closed touchscreen chambers reduced variability due to the experimenter to a minimum.

### Place Learning (PL) and Reversal Learning (RL) Task

The start of the IntelliCage experiment (IntelliCage and IntelliCagePlus 3.2.8 software: New Behavior, TSE Systems, Bad Homburg, Germany) in female mice was scheduled around the time when male mice entered the proper PAL task. Thus, males and females were largely age-matched during the last phase of the behavioral test battery (27 weeks old, resp. 22 weeks on diet). We chose not to test males in the IntelliCage setup, because males are more prone to show aggressive behavior and hierarchical fighting, potentially resulting in injuries due to the housing of male mice in large groups. The IntelliCage tasks of learning ability cover a broad cognitive spectrum by combining the analysis of spatial memory with operant conditioning [43] and provide the advantage of being both home cage and behavioral test during the time of the experiment. Animals from all experimental groups lived together in the special cage for the period of about 7 weeks. Due to this mixed group housing, the experimental diets were substituted by standard maintenance chow (ad libitum) for the duration of this behavioral test. Each apparatus had the capacity to house and detect up to 16 mice simultaneously. The experiment started with a habituation period of 1 week, followed by a pre-training phase on nose poke behavior in corners for water access for 1–2 weeks. During the following week, the animals were habituated to the two defined drinking sessions per day (5–7 a.m.; 7–9 p.m.). In the PL, only one corner per mouse yielded water access in response to nose pokes during drinking sessions (~2 weeks). Motorized doors, controlled by radio-frequency identification (RFID) transponders, opened when a mouse was detected in its adequate corner. In the RL, a different corner was designated as correct (2 weeks). Visits to the correct corners were analyzed for PL and RL. We did not weigh the animals for the duration of the experiment to avoid interference with the automated behavior recording; instead, we visually observed the mice for any sign of deficiency. The IntelliCage enabled a high throughput cognitive investigation of mice, while stress due to human intervention was reduced to a minimum.

### Sample Collection

Blood was taken by carrying out a puncture of the facial vein using 5 mm Goldenrod animal lancets (MEDIpoint, Mineola, NY, USA). A maximum volume of 170 µL per 25 g mouse according to animal welfare guidelines (GV-SOLAS) was collected in serum tubes containing a clotting factor to accelerate coagulation in the subsequent 15–30 min (Sarstedt Microvette 200 Z, Nümbrecht, Germany). The tubes were centrifuged at 3200× *g* for another 15 min at 4 °C and subsequently frozen on dry ice. For 24-h urine sampling, mice were placed into metabolic cages (Tecniplast, Hohenpeissenberg, Germany). Absolute urine volumes were documented for subsequent calculations. In order to harvest the brains, the animals were deeply anaesthetized by injecting a mixture of 200 mg/kg (body weight) ketamine (Vétoquinol GmbH, Ismaning, Germany) and 10 mg/kg (body weight) xylazine (Bayer Health Care, Leverkusen, Germany) intraperitoneally. After cessation of reflexes, blood was taken cardially and treated as described before. Mice were then perfused transcardially with 0.1 M phosphate-buffered saline (PBS) followed by 4% paraformaldehyde (Medite, Burgdorf, Germany). Brains were removed and postfixed in the same fixative for another three days followed by a stepwise dehydration in increasing ethanol concentrations (Medite) and xylene steps (Medite). Brains were then embedded in paraffin (Medite) in a heated embedding station (Thermo Fisher, Frankfurt am Main, Germany) and cut with a microtome (Thermo Fisher). 10 µm thick sections were retrieved from three different positions of the animals’ brains: −1.2, −1.7 and −2.2 posterior to bregma [81]. Data of the three positions were pooled because of absent statistically significant differences. Finally, the sections were mounted on glass slides (Klinipath, Typograaf, Netherlands).

### Biochemical Analysis

The determination of homocysteic acid (HCA) was performed as recently described in detail [36] with minor modifications, as the method was originally validated for the analysis of human serum and urine. Briefly, HCA was determined in murine serum and urine using a combination of protein precipitation and solid phase extraction for sample preparation followed by an LC–MS/MS analysis using a combination of a HILIC separation and tandem mass spectrometry. Samples were processed as previously described [36] by adding formic acid followed by protein precipitation using cooled acetonitrile. Samples were vortexed, centrifuged and loaded onto conditioned tables (Strata X AW SPE columns (33 µm, 30 mg / 1 mL, Phenomenex, Aschaffenburg, Germany) using the automated sample preparation system Extrahera (Biotage, Uppsala, Sweden). After washing the cartridges using water, methanol and a mixture of acetonitrile and aqueous ammonium hydroxide solution, HCA was eluted using two times a mixture of methanol and aqueous ammonium hydroxide solution. The eluate was dried and reconstituted by adding ammonium acetate solution and acetonitrile separately. Afterwards, the samples were injected into the LC-MS/MS system. The LC-MS/MS system consisted of a triple quadrupole mass spectrometer QTRAP 6500+ (Sciex, Darmstadt, Germany) equipped with a Turbo Ion Spray source operated in negative electrospray ionization mode and an Agilent 1290 Infinity LC-system with binary HPLC pump, column oven and autosampler (Agilent, Waldbronn, Germany). The chromatographic separation was performed using a Luna 3 µm HILIC 200 Å 100 × 2 mm column in combination with a KrudKatcher in-line filter (both Phenomenex, Aschaffenburg, Germany). Data acquisition was done using Analyst Software 1.6.3 and quantification was performed with MultiQuant Software 3.0.2 (both Sciex, Darmstadt, Germany), employing the internal standard method. Calibration curves were calculated by linear regression with 1/x weighting. Acceptance criteria and quality assurance measures have been applied as previously described [36].

The determination of homocysteine (HCys) was performed using protein precipitation in combination with LC–MS/MS. Briefly, 20 µL of serum or urine was pipetted to a polypropylene tube and 20 µL of 15 mg/mL aqueous TCEP-solution (tris(2-carboxyethyl)phosphine), 40 µL IS working solution (500 ng/mL HCys-d4 in methanolic TCEP solution, 1 mg/mL) and 40 µL methanolic TCEP solution, 1 mg/mL were added. Afterwards, samples were vortexed, centrifuged, transferred into another polypropylene tube, and dried using nitrogen. The dried samples were reconstituted using 50 µL of water containing 10 mM ammonium acetate buffer and 10 mM acetic acid, centrifuged again and injected into the LC-MS/MS system. The same LC-MS/MS-system and acceptance criteria as described for HCA were used. However, positive electrospray ionization mode was applied and a Luna Omega 1.6 μm Polar C18 100 × 2.1 mm column in combination with a respective pre column (both Phenomenex, Aschaffenburg, Germany) was used.

### Immunohistochemical Analysis

A stepwise rehydration of the brain sections was conducted, followed by a heat-induced antigen retrieval in 10 mM citrate buffer (pH 6.0) including 0.05% Tween-20 (Sigma-Aldrich, Taufkirchen, Germany). After rinsing, sections were incubated for 5 min in 0.6% H2O2 (Sigma-Aldrich) in PBS (0.1 M; pH = 7.3) in order to block endogenous peroxidases. Sections were rinsed and incubated for 30 min in PBS containing 1% bovine serum albumin (PBS-B) and 5% normal goat serum (NGS, Sigma-Aldrich) to prevent unspecific binding of the antibody. After subsequent rinsing, sections were incubated overnight at 4 °C in PBS-B containing 1% NGS and the primary antibody (anti-human Aβ 82E1 mouse IgG MoAb 1:1000, IBL international, Hamburg, Germany). Rinsing was followed by an incubation with goat anti-mouse IgG H&L Biotin (1:1000, Abcam, Berlin, Germany) in PBS-B containing 1% NGS for one hour. Sections were rinsed followed by a 1-h incubation with avidin-biotin conjugate in PBS (ABC; Vectastain Elite ABC HRP Kit, Linaris, Dossenheim, Germany). After another rinsing step, sections were treated with 3,3′-diaminobenzidine tetrahydrochloride (DAB; Sigma-Aldrich) in water (0.2 mg/mL; pH = 7.6) for 10 min. The immunostaining was then developed by adding 50 µL H_2_O_2_ to a final concentration of 0.006%, incubating for another 10 min. The reaction was stopped by rinsing in ice-cold distilled water followed by a counterstaining using Mayer’s hematoxylin (Morphisto, Frankfurt am Main, Germany). Sections were finally dehydrated and covered with Pertex (Medite). We digitized appropriate sections using a Nicon Eclipse Ni-E microscope (Nikon Instruments Europe BV, Amsterdam, Netherlands). Whole brain images were taken at a final magnification of 100x and the area occupied by plaques in several regions of interest (ROI; Table A2) was analyzed using the color segmentation plugin (Daniel Sage, Biomedical Imaging Group, EPFL, http://bigwww.epfl.ch/sage/soft/colorsegmentation/) for ImageJ software (National Institute of Health, Bethesda, MD, USA). Only animals of the first cohort were immunohistochemically investigated in this study.

**Table A2:** Regions of interest (ROI) in different cortical and hippocampal areas.

ROI	Brain Area	Height (µm)
1	Primary somatosensory cortex (PSC)	500 × 500
2	Gyrus dentatus (GD)	275 × 275
3	CA1	275 × 275
4	CA3	275 × 275
5	CA2	275 × 275
6	Thalamic nuclei (TN)	600 × 400
7	Piriform cortex (PC)	275 × 275
8	Piriform cortex (PC)	275 × 275

### Preclinical Quality Parameters

Several aspects were considered to ensure the quality of the applied methodologies and resulting data. These points are in accordance with initiatives such as EQIPD (“European quality in preclinical data”; https://quality-preclinical-data.eu/). The aim of EQIPD is broadly to implement various quality improving measures in order to enhance the reproducibility of preclinical data [63]. In the present study, we performed a power calculation to estimate the needed group size (http://www.biomath.info/power/). The resulting total amount of 112 animals was tested in two consecutive cohorts. Nine animals were lost during the course of the whole study. In terms of translatability, we have decided to include both male and female animals in the experiments, since Alzheimer’s disease affects both sexes in the clinical context, with a higher rate in women than in men [48]. In general, female animals are largely underrepresented in neuroscience research [82]. Randomization was applied at several stages along the study course. Mice were initially allocated to the home cages according to a random list (https://www.random.org/) and target holes in the Barnes maze were set randomly. Besides, drinking corners in the IntelliCages as well as the stimulus mice in the social interaction test were also assigned randomly. A within-cage randomization between groups was not applicable in this case because every mouse matched strictly to its adequate experimental diet. All animals were regularly pre-handled and transferred to the experimental rooms at least half an hour before behavioral analysis. Blinding of the experimenter in order to prevent detection bias was not performed here, because in all behavioral tests automated outcome assessment was applied (via EthoVision XT, IntelliCage and Touchsreen software). However, blinding was performed during the immunohistochemical analysis. Here, a second experimenter marked the ROI in the images without being aware of animal ID or experimental group. Furthermore, an automated animal management software as well as an electronic lab-book were used throughout the study. Standard operating procedures had been written prior to the experimental procedures.

## References

[1] (2019). World Alzheimer Report 2019: Attitudes to Dementia.

[2] Calsolaro V., Antognoli R., Okoye C., Monzani F. (2019). The Use of Antipsychotic Drugs for Treating Behavioral Symptoms in Alzheimer’s Disease. Front. Pharmacol..

[3] Sasaguri H., Nilsson P., Hashimoto S., Nagata K., Saito T., De Strooper B., Hardy J., Vassar R., Winblad B., Saido T.C. (2017). APP mouse models for Alzheimer’s disease preclinical studies. EMBO J..

[4] Hara Y., McKeehan N., Fillit H.M. (2019). Translating the biology of aging into novel therapeutics for Alzheimer disease. Neurology.

[5] Sharma P., Srivastava P., Seth A., Tripathi P.N., Banerjee A.G., Shrivastava S.K. (2019). Comprehensive review of mechanisms of pathogenesis involved in Alzheimer’s disease and potential therapeutic strategies. Prog. Neurobiol..

[6] Masters C.L., Simms G., Weinman N.A., Multhaup G., McDonald B.L., Beyreuther K. (1985). Amyloid plaque core protein in Alzheimer disease and Down syndrome. Proc. Natl. Acad. Sci. USA.

[7] Selkoe D.J., Hardy J. (2016). The amyloid hypothesis of Alzheimer’s disease at 25 years. EMBO Mol. Med..

[8] Bateman R.J., Xiong C., Benzinger T.L.S., Fagan A.M., Goate A., Fox N.C., Marcus D.S., Cairns N.J., Xie X., Blazey T.M. (2012). Clinical and Biomarker Changes in Dominantly Inherited Alzheimer’s Disease. N. Engl. J. Med..

[9] Saito T., Matsuba Y., Mihira N., Takano J., Nilsson P., Itohara S., Iwata N., Saido T.C. (2014). Single App knock-in mouse models of Alzheimer’s disease. Nat. Neurosci..

[10] Zhao G., He F., Wu C., Li P., Li N., Deng J., Zhu G., Ren W., Peng Y. (2018). Betaine in Inflammation: Mechanistic Aspects and Applications. Front. Immunol..

[11] Clarke R., Smith A.D., Jobst K.A., Refsum H., Sutton L., Ueland P.M. (1998). Folate, Vitamin B12, and Serum Total Homocysteine Levels in Confirmed Alzheimer Disease. Arch. Neurol..

[12] Isobe C., Murata T., Sato C., Terayama Y. (2005). Increase of total homocysteine concentration in cerebrospinal fluid in patients with Alzheimer’s disease and Parkinson’s disease. Life Sci..

[13] Seshadri S., Beiser A., Selhub J., Jaques P., Roseberg I.H., D’Agostino R.B., Wilson P.W.F., Wolf P.A. (2002). Plasma Homocysteine As a Risk Factor for Dementia and Alzheimer’s Disease. N. Engl. J. Med..

[14] Nurk E., Refsum H., Tell G.S., Engedal K., Vollset S.E., Ueland P.M., Nygaard H.A., Smith A.D. (2005). Plasma total homocysteine and memory in the elderly: The Hordaland homocysteine study. Ann. Neurol..

[15] Morris M.S. (2003). Homocysteine and Alzheimer’s disease. Lancet Neurol..

[16] Smith A.D., Smith S.M., de Jager C.A., Whitbread P., Johnston C., Agacinski G., Oulhaj A., Bradley K.M., Jacoby R., Refsum H. (2010). Homocysteine-Lowering by B Vitamins Slows the Rate of Accelerated Brain Atrophy in Mild Cognitive Impairment: A Randomized Controlled Trial. PLoS ONE.

[17] Douaud G., Refsum H., de Jager C.A., Jacoby R., Nichols T.E., Smith S.M., Smith A.D. (2013). Preventing Alzheimer’s disease-related gray matter atrophy by B-vitamin treatment. Proc. Natl. Acad. Sci. USA.

[18] Kennedy D. (2016). B Vitamins and the Brain: Mechanisms, Dose and Efficacy—A Review. Nutrients.

[19] McMahon J.A., Green T.J., Skeaff C.M., Knight R.G., Mann J.I., Williams S.M. (2006). A Controlled Trial of Homocysteine Lowering and Cognitive Performance. N. Engl. J. Med..

[20] Tabet N., Rafi H., Weaving G., Lyons B., Iversen S.A. (2006). Behavioural and psychological symptoms of Alzheimer type dementia are not correlated with plasma homocysteine concentration. Dement. Geriatr. Cogn. Disord..

[21] Wald D.S., Kasturiratne A., Simmonds M. (2010). Effect of Folic Acid, with or without Other B Vitamins, on Cognitive Decline: Meta-Analysis of Randomized Trials. Am. J. Med..

[22] Clarke R., Bennett D., Parish S., Lewington S., Skeaff M., Eussen S.J.P.M., Lewerin C., Stott D.J., Armitage J., Hankey G.J. (2014). Effects of homocysteine lowering with B vitamins on cognitive aging: Meta-analysis of 11 trials with cognitive data on 22,000 individuals. Am. J. Clin. Nutr..

[23] Smith A.D., Refsum H., Bottiglieri T., Fenech M., Hooshmand B., McCaddon A., Miller J.W., Rosenberg I.H., Obeid R. (2018). Homocysteine and Dementia: An International Consensus Statement. J. Alzheimers Dis..

[24] Smith A.D., Refsum H. (2016). Homocysteine, B Vitamins, and Cognitive Impairment. Annu. Rev. Nutr..

[25] Grimm M.O.W., Michaelson D.M., Hartmann T. (2017). Omega-3 fatty acids, lipids, and apoE lipidation in Alzheimer’s disease: A rationale for multi-nutrient dementia prevention. J. Lipid Res..

[26] Oulhaj A., Jernerén F., Refsum H., Smith A.D., de Jager C.A. (2016). Omega-3 Fatty Acid Status Enhances the Prevention of Cognitive Decline by B Vitamins in Mild Cognitive Impairment. J. Alzheimers Dis..

[27] McCleery J., Abraham R.P., Denton D.A., Rutjes A.W.S., Chong L.-Y., Al-Assaf A.S., Griffith D.J., Rafeeq S., Yaman H., Malik M.A. (2018). Vitamin and mineral supplementation for preventing dementia or delaying cognitive decline in people with mild cognitive impairment. Cochrane Database Syst. Rev..

[28] Diaz-Arrastia R. (2000). Homocysteine and Neurologic Disease. Arch. Neurol..

[29] Obeid R., Herrmann W. (2006). Mechanisms of homocysteine neurotoxicity in neurodegenerative diseases with special reference to dementia. FEBS Lett..

[30] Lipton S.A., Kim W.-K., Choi Y.-B., Kumar S., D’Emilia D.M., Rayudu P.V., Arnelle D.R., Stamler J.S. (1997). Neurotoxicity associated with dual actions of homocysteine at the N-methyl-D-aspartate receptor. Proc. Natl. Acad. Sci. USA.

[31] Kim J.P., Koh J., Choi D.W. (1987). l-Homocysteate is a potent neurotoxin on cultured cortical neurons. Brain Res..

[32] Sommer S., Hunzinger C., Schillo S., Klemm M., Biefang-Arndt K., Schwall G., Pütter S., Hoelzer K., Schroer K., Stegmann W. (2004). Molecular Analysis of Homocysteic Acid-Induced Neuronal Stress. J. Proteome Res..

[33] Görtz P., Hoinkes A., Fleischer W., Otto F., Schwahn B., Wendel U., Siebler M. (2004). Implications for hyperhomocysteinemia: Not homocysteine but its oxidized forms strongly inhibit neuronal network activity. J. Neurol. Sci..

[34] Vladychenskaya E.A., Tyulina O.V., Boldyrev A.A. (2006). Effect of Homocysteine and Homocysteic Acid on Glutamate Receptors on Rat Lymphocytes. Bull. Exp. Biol. Med. Vol..

[35] Jansen D., Zerbi V., Arnoldussen I.A.C., Wiesmann M., Rijpma A., Fang X.T., Dederen P.J., Mutsaers M.P.C., Broersen L.M., Lütjohann D. (2013). Effects of Specific Multi-Nutrient Enriched Diets on Cerebral Metabolism, Cognition and Neuropathology in AβPPswe-PS1dE9 Mice. PLoS ONE.

[36] Gurke R., Schmidt D., Thomas D., Fleck S.C., Geisslinger G., Ferreirós N. (2019). A validated LC–MS/MS method for the determination of homocysteic acid in biological samples. J. Pharm. Biomed. Anal..

[37] Bolivar V.J. (2009). Intrasession and intersession habituation in mice: From inbred strain variability to linkage analysis. Neurobiol. Learn. Mem..

[38] Gawel K., Gibula E., Marszalek-Grabska M., Filarowska J., Kotlinska J.H. (2019). Assessment of spatial learning and memory in the Barnes maze task in rodents—Methodological consideration. Naunyn. Schmiedebergs. Arch. Pharmacol..

[39] Kaidanovich-Beilin O., Lipina T., Vukobradovic I., Roder J., Woodgett J.R. (2011). Assessment of Social Interaction Behaviors. J. Vis. Exp..

[40] Moy S.S., Nadler J.J., Perez A., Barbaro R.P., Johns J.M., Magnuson T.R., Piven J., Crawley J.N. (2004). Sociability and preference for social novelty in five inbred strains: An approach to assess autistic-like behavior in mice. Genes Brain Behav..

[41] Nithianantharajah J., McKechanie A.G., Stewart T.J., Johnstone M., Blackwood D.H., St Clair D., Grant S.G.N., Bussey T.J., Saksida L.M. (2015). Bridging the translational divide: Identical cognitive touchscreen testing in mice and humans carrying mutations in a disease-relevant homologous gene. Sci. Rep..

[42] Jacob S., Davies G., De Bock M., Hermans B., Wintmolders C., Bottelbergs A., Borgers M., Theunis C., Van Broeck B., Manyakov N.V. (2019). Neural oscillations during cognitive processes in an App knock-in mouse model of Alzheimer’s disease pathology. Sci. Rep..

[43] Voikar V., Krackow S., Lipp H.-P., Rau A., Colacicco G., Wolfer D.P. (2018). Automated dissection of permanent effects of hippocampal or prefrontal lesions on performance at spatial, working memory and circadian timing tasks of C57BL/6 mice in IntelliCage. Behav. Brain Res..

[44] Krackow S., Vannoni E., Codita A., Mohammed A.H., Cirulli F., Branchi I., Alleva E., Reichelt A., Willuweit A., Voikar V. (2010). Consistent behavioral phenotype differences between inbred mouse strains in the IntelliCage. Genes Brain Behav..

[45] Agrawal A., Ilango K., Singh P.K., Karmakar D., Singh G.P.I., Kumari R., Dubey G.P. (2015). Age dependent levels of plasma homocysteine and cognitive performance. Behav. Brain Res..

[46] Zahs K.R., Ashe K.H. (2010). ‘Too much good news’—Are Alzheimer mouse models trying to tell us how to prevent, not cure, Alzheimer’s disease?. Trends Neurosci..

[47] Perneczky R., Drzezga A., Diehl-Schmid J., Li Y., Kurz A. (2007). Gender differences in brain reserve. J. Neurol..

[48] Mielke M., Vemuri P., Rocca W. (2014). Clinical epidemiology of Alzheimer’ disease: Assessing sex and gender differences. Clin. Epidemiol..

[49] Sakakibara Y., Sekiya M., Saito T., Saido T.C., Iijima K.M. (2019). Amyloid-β plaque formation and reactive gliosis are required for induction of cognitive deficits in App knock-in mouse models of Alzheimer’s disease. BMC Neurosci..

[50] Mehla J., Lacoursiere S.G., Lapointe V., McNaughton B.L., Sutherland R.J., McDonald R.J., Mohajerani M.H. (2019). Age-dependent behavioral and biochemical characterization of single APP knock-in mouse (APPNL-G-F/NL-G-F) model of Alzheimer’s disease. Neurobiol. Aging.

[51] Latif-Hernandez A., Shah D., Craessaerts K., Saido T., Saito T., De Strooper B., Van der Linden A., D’Hooge R. (2019). Subtle behavioral changes and increased prefrontal-hippocampal network synchronicity in APPNL−G−F mice before prominent plaque deposition. Behav. Brain Res..

[52] Whyte L.S., Hemsley K.M., Lau A.A., Hassiotis S., Saito T., Saido T.C., Hopwood J.J., Sargeant T.J. (2018). Reduction in open field activity in the absence of memory deficits in the App NL−G−F knock-in mouse model of Alzheimer’s disease. Behav. Brain Res..

[53] Sakakibara Y., Sekiya M., Saito T., Saido T.C., Iijima K.M. (2018). Cognitive and emotional alterations in App knock-in mouse models of Aβ amyloidosis. BMC Neurosci..

[54] Masuda A., Kobayashi Y., Kogo N., Saito T., Saido T.C., Itohara S. (2016). Cognitive deficits in single App knock-in mouse models. Neurobiol. Learn. Mem..

[55] Jankowsky J.L., Zheng H. (2017). Practical considerations for choosing a mouse model of Alzheimer’s disease. Mol. Neurodegener..

[56] Sudduth T.L., Powell D.K., Smith C.D., Greenstein A., Wilcock D.M. (2013). Induction of Hyperhomocysteinemia Models Vascular Dementia by Induction of Cerebral Microhemorrhages and Neuroinflammation. J. Cereb. Blood Flow Metab..

[57] Teri L., Ferretti L.E., Gibbons L.E., Logsdon R.G., McCurry S.M., Kukull W.A., McCormick W.C., Bowen J.D., Larson E.B. (1999). Anxiety in Alzheimer’s Disease: Prevalence and Comorbidity. J. Gerontol. Ser. A Biol. Sci. Med. Sci..

[58] Fuso A., Nicolia V., Cavallaro R.A., Ricceri L., D’Anselmi F., Coluccia P., Calamandrei G., Scarpa S. (2008). B-vitamin deprivation induces hyperhomocysteinemia and brain S-adenosylhomocysteine, depletes brain S-adenosylmethionine, and enhances PS1 and BACE expression and amyloid-β deposition in mice. Mol. Cell. Neurosci..

[59] Zhang C.-E., Wei W., Liu Y.-H., Peng J.-H., Tian Q., Liu G.-P., Zhang Y., Wang J.-Z. (2009). Hyperhomocysteinemia Increases β-Amyloid by Enhancing Expression of γ-Secretase and Phosphorylation of Amyloid Precursor Protein in Rat Brain. Am. J. Pathol..

[60] Kruman I.I., Kumaravel T.S., Lohani A., Pedersen W.A., Cutler R.G., Kruman Y., Haughey N., Lee J., Evans M., Mattson M.P. (2002). Folic acid deficiency and homocysteine impair DNA repair in hippocampal neurons and sensitize them to amyloid toxicity in experimental models of Alzheimer’s disease. J. Neurosci..

[61] Bernardo A., McCord M., Troen A.M., Allison J.D., McDonald M.P. (2007). Impaired spatial memory in APP-overexpressing mice on a homocysteinemia-inducing diet. Neurobiol. Aging.

[62] Troen A.M., Shea-Budgell M., Shukitt-Hale B., Smith D.E., Selhub J., Rosenberg I.H. (2008). B-vitamin deficiency causes hyperhomocysteinemia and vascular cognitive impairment in mice. Proc. Natl. Acad. Sci. USA.

[63] Bespalov A., Steckler T., Skolnick P. (2019). Be positive about negatives–recommendations for the publication of negative (or null) results. Eur. Neuropsychopharmacol..

[64] Refsum H., Smith A.D., Ueland P.M., Nexo E., Clarke R., McPartlin J., Johnston C., Engbaek F., Schneede J., McPartlin C. (2004). Facts and Recommendations about Total Homocysteine Determinations: An Expert Opinion. Clin. Chem..

[65] Sinha M., Saha A., Basu S., Pal K., Chakrabarti S. (2010). Aging and antioxidants modulate rat brain levels of homocysteine and dehydroepiandrosterone sulphate (DHEA-S): Implications in the pathogenesis of Alzheimer’s disease. Neurosci. Lett..

[66] Ueland P.M., Nygård O., Vollset S.E., Refsum H. (2001). The Hordaland Homocysteine Studies. Lipids.

[67] Ernest S., Hosack A., O’Brien W.E., Rosenblatt D.S., Nadeau J.H. (2005). Homocysteine levels in A/J and C57BL/6J mice: Genetic, diet, gender, and parental effects. Physiol. Genom..

[68] Zhuo J.-M., Praticò D. (2010). Severe In Vivo Hyper-Homocysteinemia is not Associated with Elevation of Amyloid-β Peptides in the Tg2576 Mice. J. Alzheimers Dis..

[69] Hasegawa T., Mikoda N., Kitazawa M., LaFerla F.M. (2010). Treatment of Alzheimer’s Disease with Anti-Homocysteic Acid Antibody in 3xTg-AD Male Mice. PLoS ONE.

[70] Janssen C.I.F., Zerbi V., Mutsaers M.P.C., de Jong B.S.W., Wiesmann M., Arnoldussen I.A.C., Geenen B., Heerschap A., Muskiet F.A.J., Jouni Z.E. (2015). Impact of dietary n-3 polyunsaturated fatty acids on cognition, motor skills and hippocampal neurogenesis in developing C57BL/6J mice. J. Nutr. Biochem..

[71] Wiesmann M., Zerbi V., Jansen D., Haast R., Lütjohann D., Broersen L.M., Heerschap A., Kiliaan A.J. (2016). A Dietary Treatment Improves Cerebral Blood Flow and Brain Connectivity in Aging apoE4 Mice. Neural Plast..

[72] Arendash G.W., Jensen M.T., Salem N., Hussein N., Cracchiolo J., Dickson A., Leighty R., Potter H. (2007). A diet high in omega-3 fatty acids does not improve or protect cognitive performance in Alzheimer’s transgenic mice. Neuroscience.

[73] Shah R.C., Kamphuis P.J., Leurgans S., Swinkels S.H., Sadowsky C.H., Bongers A., Rappaport S.A., Quinn J.F., Wieggers R.L., Scheltens P. (2013). The S-Connect study: Results from a randomized, controlled trial of Souvenaid in mild-to-moderate Alzheimer’s disease. Alzheimers Res. Ther..

[74] Scheltens N.M.E., Briels C.T., Yaqub M., Barkhof F., Boellaard R., van der Flier W.M., Schwarte L.A., Teunissen C.E., Attali A., Broersen L.M. (2019). Exploring effects of Souvenaid on cerebral glucose metabolism in Alzheimer’s disease. Alzheimers Dement. Transl. Res. Clin. Interv..

[75] Panza F., Lozupone M., Logroscino G., Imbimbo B.P. (2019). A critical appraisal of amyloid-β-targeting therapies for Alzheimer disease. Nat. Rev. Neurol..

[76] Aizenstein H.J., Nebes R.D., Saxton J.A., Price J.C., Mathis C.A., Tsopelas N.D., Ziolko S.K., James J.A., Snitz B.E., Houck P.R. (2008). Frequent Amyloid Deposition Without Significant Cognitive Impairment Among the Elderly. Arch. Neurol..

[77] Kaplon H., Muralidharan M., Schneider Z., Reichert J.M. (2020). Antibodies to watch in 2020. MAbs.

[78] Tucker L.B., McCabe J.T. (2017). Behavior of Male and Female C57BL/6J Mice Is More Consistent with Repeated Trials in the Elevated Zero Maze than in the Elevated Plus Maze. Front. Behav. Neurosci..

[79] Harrison F.E., Reiserer R.S., Tomarken A.J., McDonald M.P. (2006). Spatial and nonspatial escape strategies in the Barnes maze. Learn. Mem..

[80] Talpos J.C., Winters B.D., Dias R., Saksida L.M., Bussey T.J. (2009). A novel touchscreen-automated paired-associate learning (PAL) task sensitive to pharmacological manipulation of the hippocampus: A translational rodent model of cognitive impairments in neurodegenerative disease. Psychopharmacology.

[81] Paxinos G., Franklin K.B.J. (2013). The Mouse Brain in Stereotaxic Coordinates.

[82] Beery A.K. (2018). Inclusion of females does not increase variability in rodent research studies. Curr. Opin. Behav. Sci..

